# Conserved residues in the extracellular loop 2 regulate *Stachel*-mediated activation of ADGRG2

**DOI:** 10.1038/s41598-021-93577-y

**Published:** 2021-07-07

**Authors:** Abanoub A. Gad, Pedram Azimzadeh, Nariman Balenga

**Affiliations:** 1 Department of Surgery, University of Maryland School of Medicine, Baltimore, MD USA; 2 Graduate Program in Life Sciences, University of Maryland, Baltimore, MD USA; 3 Department of Pharmacology, University of Maryland School of Medicine, Baltimore, MD USA; 4 Molecular and Structural Biology program at University of Maryland Marlene and Stewart Greenebaum NCI Comprehensive Cancer Center, Baltimore, MD USA; 5 Department of Surgery and Pharmacology, University of Maryland School of Medicine, 655 W. Baltimore Street, Room 10-027, Baltimore, MD 21201 USA

**Keywords:** Receptor pharmacology, Cell signalling

## Abstract

Cleavage and dissociation of a large N-terminal fragment and the consequent unmasking of a short sequence (*Stachel*) remaining on the N-terminus have been proposed as mechanisms of activation of some members of the adhesion G protein-coupled receptor (aGPCR) family. However, the identity of residues that play a role in the activation of aGPCRs by the cognate *Stachel* remains largely unknown. Protein sequence alignments revealed a conserved stretch of residues in the extracellular loop 2 (ECL2) of all 33 members of the aGPCR family. ADGRG2, an orphan aGPCR, plays a major role in male fertility, Ewing sarcoma cell proliferation, and parathyroid cell function. We used ADGRG2 as a model aGPCR and generated mutants of the conserved residues in the ECL2 via site-directed mutagenesis. We show that tryptophan and isoleucine in the ECL2 are essential for receptor stability and surface expression in the HEK293 cells. By adjusting the receptor surface expression levels, we show that mutation of these residues of ECL2 ablates the *Stachel*-mediated activation of multiple signaling pathways of ADGRG2. This study provides a novel understanding of the role of the ECL2 in *Stachel*-mediated signaling and degradation of ADGRG2, which may lay the foundation for the rational design of therapeutics to target aGPCRs.

## Introduction

G protein-coupled receptors (GPCRs) play fundamental roles in various cellular processes such as proliferation, metabolism, hormone secretion, contraction, and neurotransmission^1^. The signaling pathways of GPCRs via G proteins and β-arrestins, and their trafficking trajectories have been studied for several decades^2,3^. This in combination with recent advances in our understanding of GPCR structures has formed a strong foundation for rational drug design to target this largest superfamily of surface receptors^4,5^.

Recent genomic and model organism studies have revealed the versatile roles that the adhesion GPCRs (aGPCRs), the second-largest family of GPCRs, play in endocrine, nervous and immune systems^6^. aGPCRs have structural differences with other classes of GPCRs, namely autoproteolysis at a GPCR proteolysis site (GPS) and formation of a two-segmented receptor with a large N-terminal fragment (NTF) and a C-terminal fragment (CTF). The CTF includes the C-terminus, seven-transmembrane helical domains (TM1-7), extracellular loops (ECL1-3), intracellular loops (ICL1-3), and a short extracellular sequence on the N-terminus just before the TM1. Many studies^7–10^, including our recent reports^11,12^, have shown that this short remaining peptide, known as *Stachel* sequence, can activate aGPCRs and might be one of the mechanisms of activation in physiological and pathological states. NTF-dissociating molecular partners can potentially unmask the *Stachel* sequence for its binding to the cognate aGPCR. These studies exploited two main tools to reveal this mechanism: (a) NTF-truncated aGPCRs that show constitutive activation of G proteins; (b) synthetic peptides resembling the *Stachel* sequence that activate aGPCRs^13,14^. Other mechanisms or modes of aGPCR activation have been reported as well. For instance, a mutant of ADGRG1 that lacks both NTF and *Stachel* was shown to retain constitutive activation of nuclear factor of activated T cells (NFAT) and recruitment of β-arrestins^14^, pointing to dual roles of NTF, shielding the *Stachel* peptide and inhibiting the intrinsic activity of the CTF. Also, a non-cleavable mutant of ADGRD1^15^ or non-cleavable wildtype forms of ADGRB1^16^ and ADGRG5^17^ retain their signaling capabilities. These studies show that aGPCRs use various structural segments, motifs, and the *Stachel* peptide to engage distinct signaling modes^18^.

We recently showed that human ADGRG2 (GPR64), an orphan aGPCR, is expressed in human parathyroid glands and regulates the signaling and function of calcium-sensing receptor^12^. We discovered that similar to other aGPCRs (ADGRG1^19^, ADGRD1^8^, ADGRG6^8^), ADGRG2 is activated by either the endogenous 15-amino acid long *Stachel* (P-15) on the N-terminus of its NTF-truncated mutant (ADGRG2-∆NTF) or the synthetic P-15^12^. We showed that the deletion of P-15, in addition to the NTF, ablates constitutive activation of Gαs and cAMP production in a mutant that starts with the proline 622 (ADGRG2-P622)^11^ and elevates receptor response to synthetic P-15. Nevertheless, the binding site of *Stachel* remains unknown among aGPCRs.

**Figure 1:**
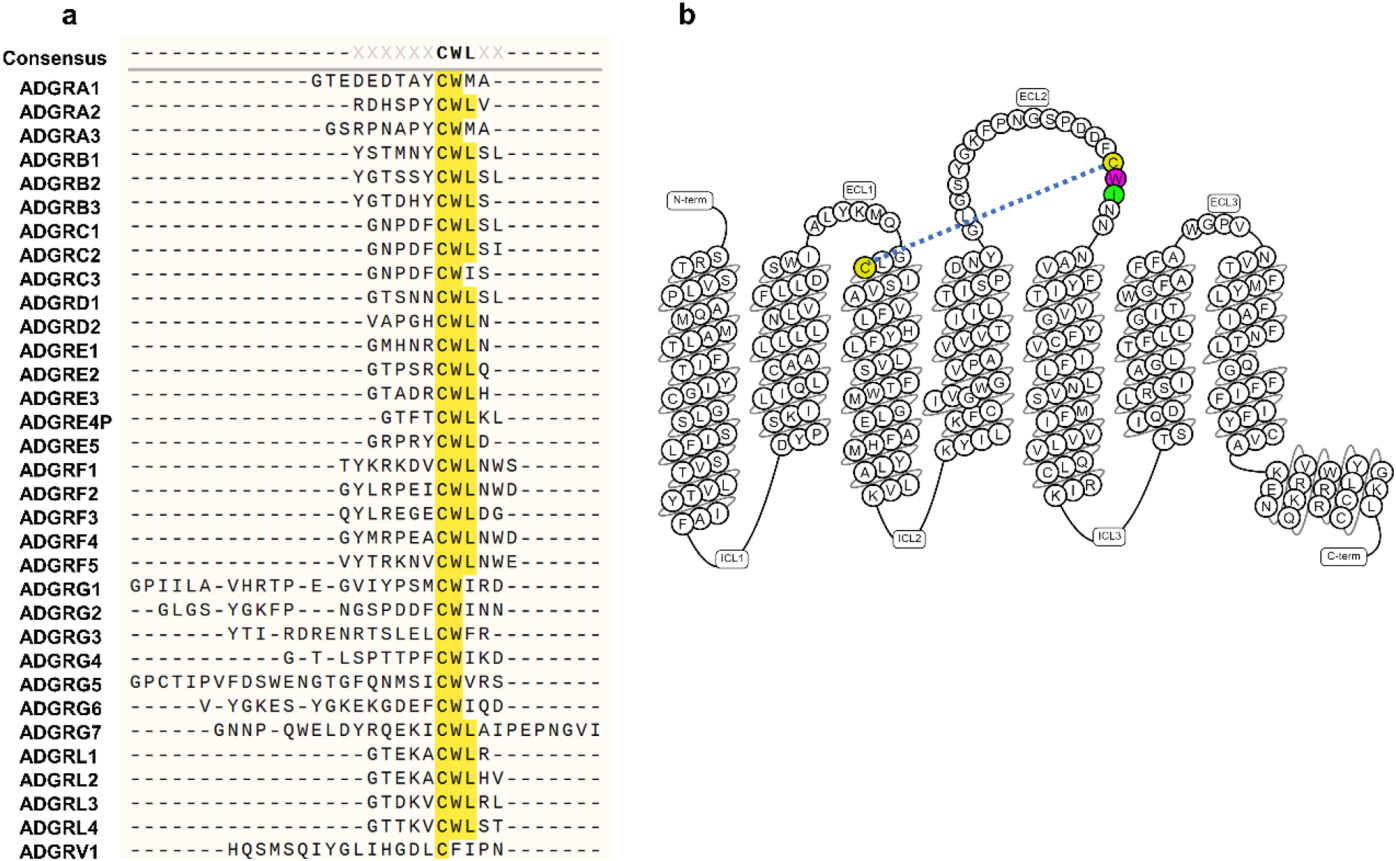
Conserved residues in the extracellular loop 2 (ECL2) of aGPCRs. (**a**) Multiple alignments of the ECL2 of all 33 members of the aGPCR family. Predicted amino acid sequences of the ECL2 for each aGPCR were derived from GPCRdb. The alignment was conducted in SnapGene software (from Insightful Science; available at www.snapgene.com) using the Clustal Omega algorithm^46^. (**b**) Snakeplot of human ADGRG2, exported from www.GPCRdb.org, showing the colored CWI motif in the ECL2. The sequences of the N-terminal fragment and C-terminus are not shown. The dashed line shows the disulfide bond between the cysteines of TM3 and ECL2.

Previous studies in other families of GPCRs revealed that some residues in the ECL2 play major roles in ligand access, receptor subtype selectivity, and activity^20–22^. Despite high degrees of diversity in the structure of ECL2 among GPCRs^23^, there is a conserved disulfide bond between the cysteines of ECL2 and TM3, which ensures receptor structural integrity. In the Secretin family (class B1), this conserved ECL2 cysteine is followed by a tryptophan residue, forming the CW motif, which is further followed by an acidic residue (aspartic or glutamic acid). The aGPCR family has the highest homology to the Secretin family. By aligning the ECL2 residues of all 33 human aGPCRs (predicted by either GPCRdb or Uniprot), we show that most of the aGPCRs have an aliphatic residue (leucine or isoleucine) after the CW motif (Fig. 1a and Supplementary Fig. 1). Whether the ECL2 plays a role in the activation of aGPCRs by *Stachel* remains poorly studied.

Here, we use ADGRG2 (P622 and ∆NTF) as a model aGPCR to investigate the role of tryptophan and isoleucine of ECL2 (Fig. 1b) in receptor activation by the *Stachel*. Our study shows that these residues not only regulate receptor activation by P-15 but also control receptor surface expression and degradation.

## Results

### Mutations in ECL2 alter the expression of ADGRG2

**Figure 2:**
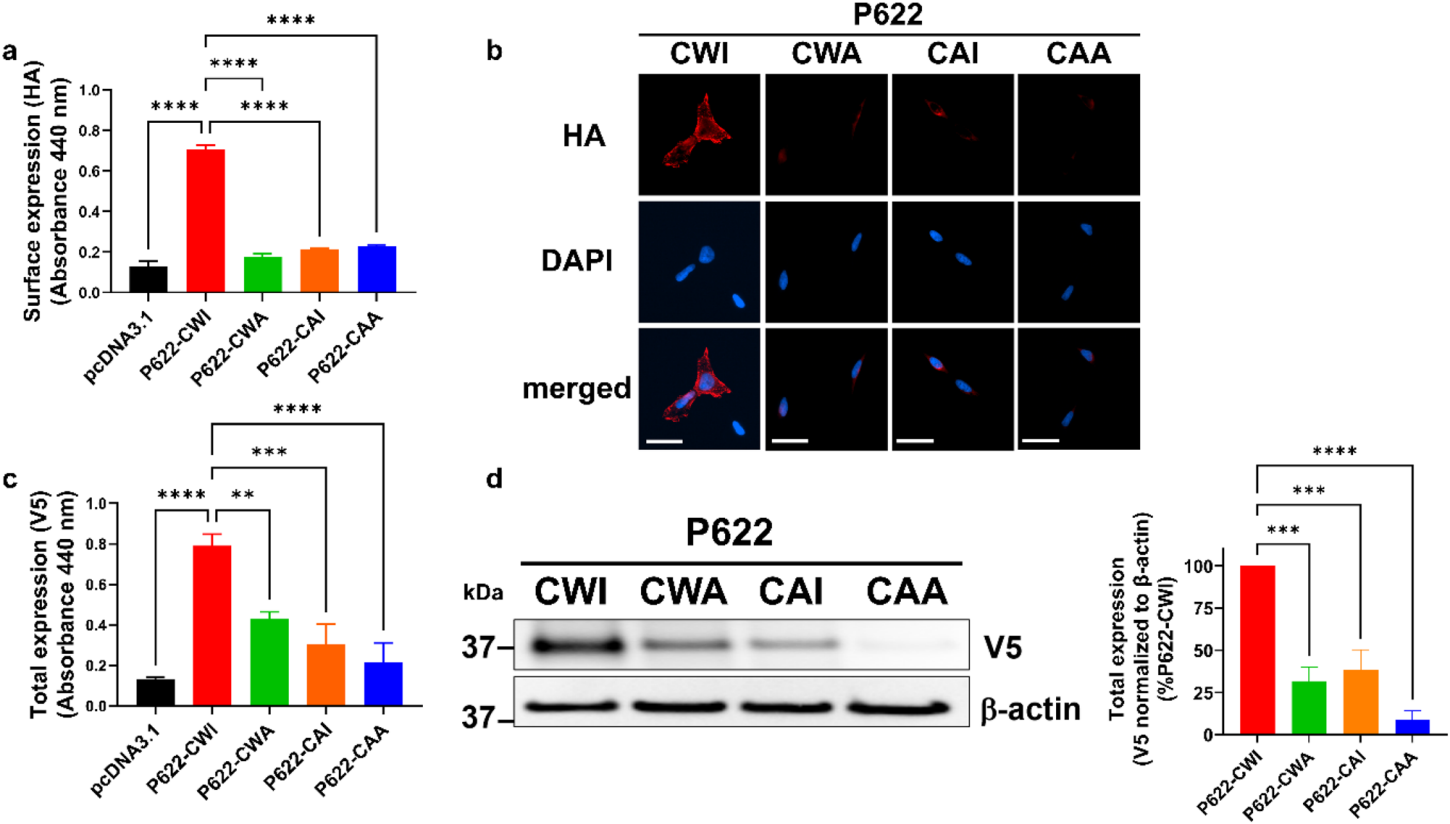
Expression of ADGRG2-P622 is regulated by the residues in the CWI motif in the ECL2. HEK cells were transfected with the same dose (50 ng for **a**–**c**; 1 µg for **d**) of plasmids expressing P622-CWI or mutants. Cell surface expression of receptors was determined by (**a**) ELISA and (**b**) immunofluorescence imaging using an antibody against the N-terminal HA-tag in non-permeabilized conditions. Nuclear counterstaining with DAPI (scale bars: 20 µm). (**c**) ELISA was used to measure the total expression by using an antibody against the C-terminal V5 tag in permeabilized conditions. (**d**) Representative blots show C-terminal V5-tagged P622-CWI and mutant receptors in total cell lysates. Densitometry data were used to normalize V5 expression to β-actin for each plasmid and are shown as a percentage of P622-CWI. Uncropped blots are provided in Supplementary Fig. 5. Data are mean ± S.E.M from a representative experiment out of 4 (for **a** and **c**, performed in quadruplicate) independent experiments. Images (**b** and **d**) are representative of 3 independent experiments. One-Way ANOVA with Dunnett’s multiple comparison test was used for statistical analyses. *P < 0.05; **P < 0.01; ***P < 0.001; ****P < 0.0001.

We transiently expressed the ADGRG2-P622 (P622-CWI) and its ECL2 mutants, P622-CWA, P622-CAI, and P622-CAA in HEK cells and used a cell-based ELISA assay to determine their surface expression via their N-terminal HA-tag. We noticed that all ECL2-mutants show significantly reduced expression on the surface in comparison to the P622-CWI receptor (Fig. 2a). This lower surface expression was confirmed by immunofluorescence imaging of receptors on the cell surface (Fig. 2b). Measuring the total expression via the V5-tag at the C-terminus by ELISA revealed that all three mutants are expressed at reduced levels compared to the P622-CWI receptor (Fig. 2c). Western blot analyses of the whole-cell lysates confirmed the reduced expression of mutants (Fig. 2d).

We have previously shown that the P622 receptor lacks the constitutive activity of the NTF-truncated (∆NTF) ADGRG2. We observed a similar reduction of expression in mutants of the CWI motif in ∆NTF receptor (∆NTF-CWA, ∆NTF-CAI, ∆NTF-CAA) compared with the ∆NTF-CWI receptor (Supplementary Fig. 2). Interestingly, mutation of P622 on residues other than the CWI motif, glycine^772^ and serine^773^, to alanine (P622-^G772A/S773A^) did not alter the total or surface expression level of the receptor (Supplementary Fig. 3a,b).

Together, these data suggest that the level of ADGRG2 is controlled by tryptophan and isoleucine in the CWI motif of the ECL2, irrespective of the activation state of the receptor.

### ECL2-mutations accelerate ADGRG2 degradation

**Figure 3:**
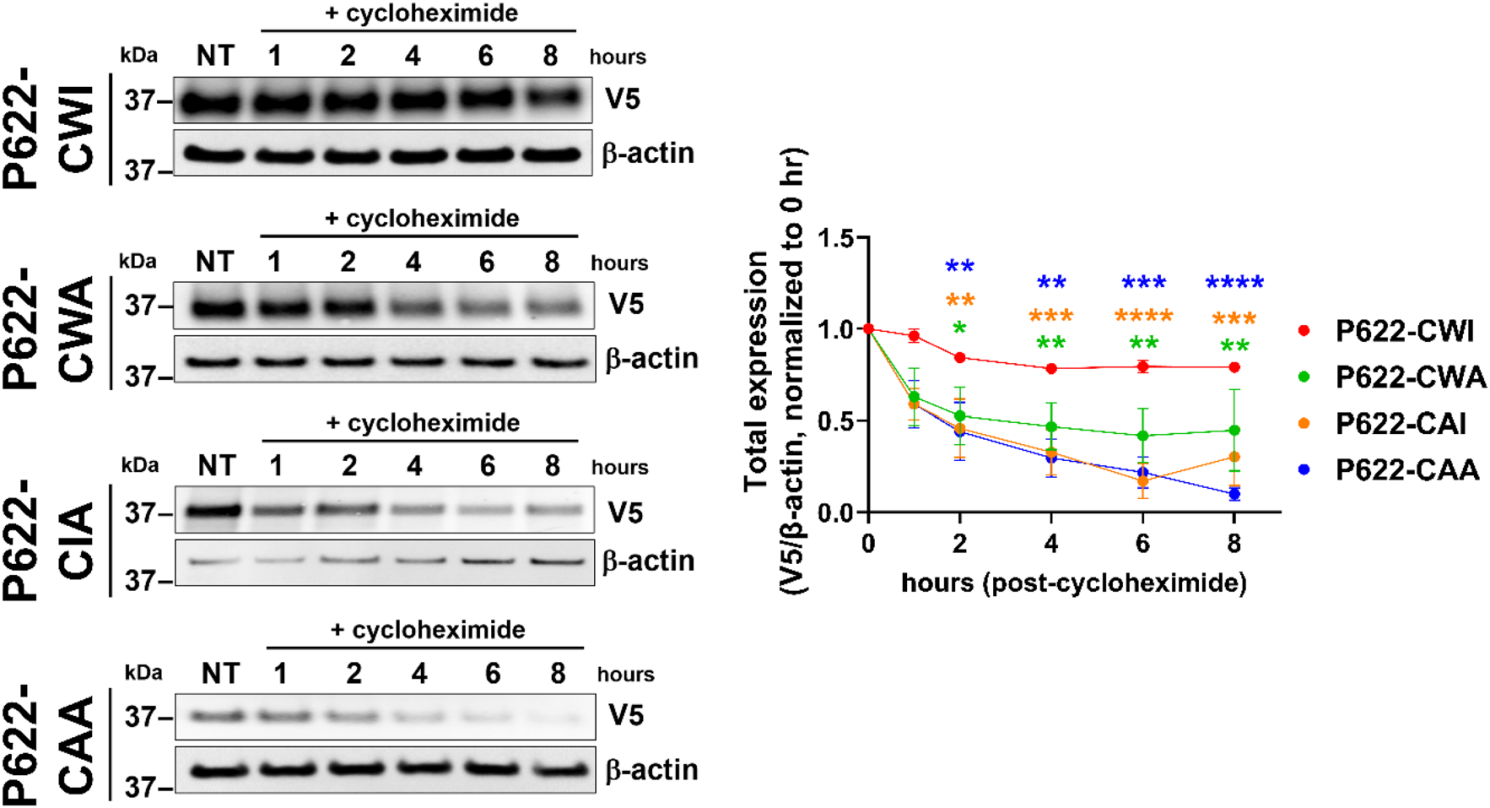
Degradation of ADGRG2-P622 is regulated by the residues in the CWI motif in the ECL2. HEK cells were transfected with the same dose of plasmids (1 µg) expressing P622-CWI or mutants. Cells were then incubated with cycloheximide (100 µg/ml) for up to 8 h and total cell lysates were run on SDS-PAGE, transferred to PVDF membranes, and probed with V5 and β-actin antibodies. Differential exposure time in the iBright Imaging system was used to make the mutants visible to facilitate the band density quantification. Representative blots show a reduction of mutant receptor levels over time. Density values of V5 were normalized to that of β-actin for each plasmid at NT (0 h). Densitometry analyses are shown on the right. Data are mean ± S.E.M from 4 independent experiments. Uncropped blots are provided in Supplementary Fig. 5. Two-Way ANOVA with Dunnett’s multiple test was used to compare protein levels at each time point to that of basal expression (NT) of respective receptors. *P < 0.05; **P < 0.01; ***P < 0.001; ****P < 0.0001.

To determine if the reduced expression is due to possible degradation of ECL2 mutants of P622, we isolated cell lysates at several time points post-incubation with a protein translation inhibitor, cycloheximide. The expression of the receptor with intact CWI motif remained substantially unchanged up to 8 h post-inhibition of translation. However, the protein level of all P622-CWA, P622-CAI, and P622-CAA was significantly reduced starting at 2 h post-inhibition and continued to diminish continuously up to 8 h (Fig. 3).

**Figure 4:**
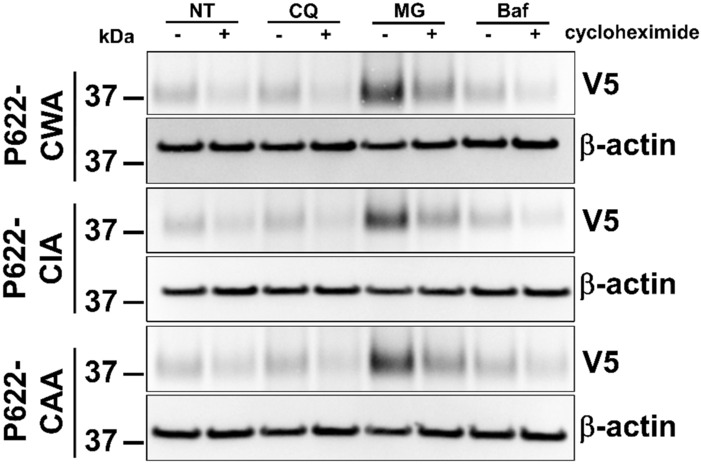
Proteasomal but not endosomal or lysosomal pathways control degradation of ECL2 mutants. (**a**) HEK cells were transfected with the same dose of plasmids (1 µg) expressing P622-CWA, P622-CAI, and P622-CAA. Cells were then pre-incubated with chloroquine (CQ; lysosomal acidification inhibitor, 25 µM), MG-132 (MG; proteasomal inhibitor, 1 µM), and bafilomycin A1 (Baf; inhibitor of v-ATPase, endosomal acidification, and late-stage vesicle maturation, 10 nM) overnight, followed by an 8-h incubation with cycloheximide (100 µg/ml). Total cell lysates were run on SDS-PAGE, transferred to PVDF membranes, and probed with V5 and β-actin antibodies. Representative blots from 3 independent experiments are shown. Uncropped blots are provided in Supplementary Fig. 5.

GPCR degradation can occur at various stages, including lysosomes and proteasomes^24^. We used inhibitors of several proteolytic processes, chloroquine (lysosomal acidification inhibitor), MG-132 (proteasomal inhibitor), and bafilomycin A1 (inhibitor of v-ATPase, endosomal acidification, and late-stage vesicle maturation) to determine the underlying mechanism of degradation of ECL2 mutants of P622. Western blotting analyses show that the expression of ECL2 mutants remained unchanged in the presence of either chloroquine or bafilomycin and cycloheximide further decreased the receptor levels (Fig. 4). However, inhibition of proteasomal degradation by MG-132 substantially increased the receptor levels, though the 8-h translation inhibition by cycloheximide in the presence of MG-132 reduced the expression to basal levels (Fig. 4).

This set of data reveals that mutation of conserved residues in the ECL2 of ADGRG2 induces receptor degradation via an unidentified mechanism, in addition to a proteasomal pathway.

### Mutation of tryptophan and isoleucine in ECL2 ablates ADGRG2-mediated induction of transcription factors

**Figure 5:**
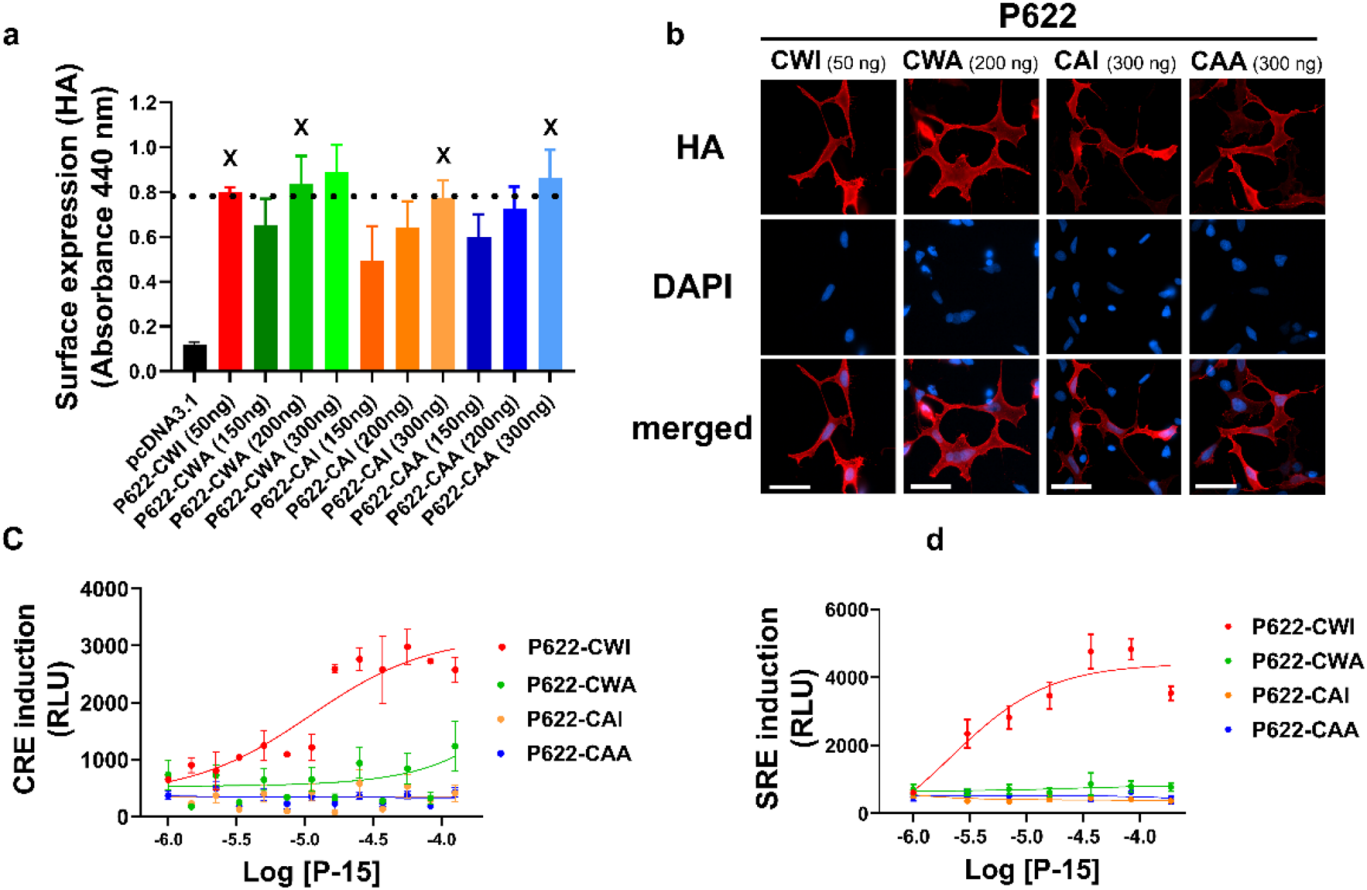
ECL2 plays a major role in ADGRG2 activation by synthetic *Stachel* peptide. (**a**) HEK cells were transfected with one dose of P622-CWI (50 ng) and different doses of mutant P622 plasmids. Cell surface expression of receptors was determined by ELISA using an antibody against the N-terminal HA-tag in non-permeabilized conditions. X denotes the amount of mutant plasmids that resulted in comparable surface expression as 50 ng P622-CWI plasmid. Data are mean ± S.E.M from a representative experiment out of 3 independent experiments performed in quadruplicate. (**b**) Cell surface expression of receptors, after transfection with the adjusted doses of plasmids, was determined by immunofluorescence imaging using an antibody against the N-terminal HA-tag in non-permeabilized conditions. Nuclear counterstaining with DAPI. Representative images from 3 independent experiments are shown (scale bars: 20 µm). (**c**, **d**) Cells were transfected with adjusted doses of either P622-CWI or mutant plasmids along with either CRE-Luc (**c**) or SRE-Luc (**d**) plasmids. After an overnight of serum starvation, cells were activated with increasing concentrations of P-15 for 5 h. Luciferase induction was measured in a luminescence-based assay. Relative light units (RLU) recorded in a luminometer are representative from 3 independent experiments performed in triplicate and are presented as mean ± S.E.M.

We have previously shown that the synthetic P-15 induces cAMP-response element binding protein (CRE) transcription factor in a luciferase reporter assay. To test whether ECL2 mutations affect receptor activation by the *Stachel* peptide despite their reduced surface expression, we adjusted the dose of the transfected plasmids so that the mutants and P622-CWI receptor reach similar surface expression levels (Fig. 5a). This was further confirmed by immunofluorescence imaging (Fig. 5b). Thereafter, for all signaling assays, we adjusted the doses of mutant and P622-CWI plasmids and supplemented the total plasmid dose with empty backbone pcDNA3.1 plasmid.

Using the CRE and SRE reporter assays, we stimulated cells expressing comparable surface levels of P622-CWI and mutant receptors with increasing concentrations of P-15 or vehicle for 5 h. The basal CRE and SRE activity was similar among P622-CWI and mutant receptors. Concentration–response curves revealed that while the P622-CWI receptor responds in a concentration-dependent manner to P-15, none of the mutants are activated by this synthetic peptide (Fig. 5c,d). Although the mutation of G^772^S^773^ to alanine in the ECL2 had no effect on the expression level, the CRE and SRE response to P-15 was reduced by 30% and 40%, respectively (Supplementary Fig. 3c,d).

**Figure 6:**
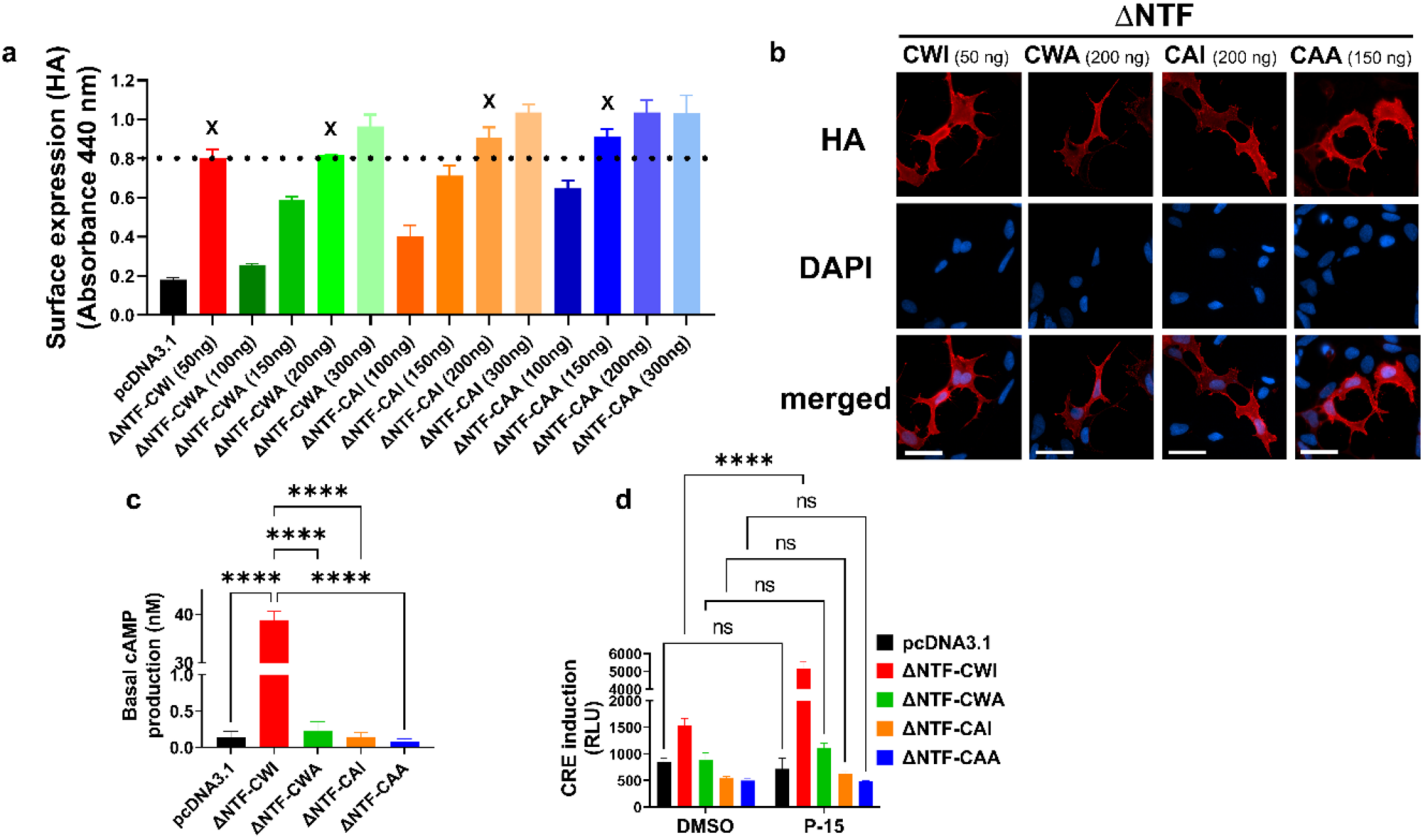
ECL2 is essential for the constitutive activity of NTF-truncated ADGRG2. (**a**) HEK cells were transfected with one dose of ∆NTF-CWI (50 ng) and different doses of mutant ∆NTF plasmids. Cell surface expression of receptors was determined by ELISA using an antibody against the N-terminal HA-tag in non-permeabilized conditions. X denotes the amount of mutant plasmids that resulted in comparable surface expression as 50 ng ∆NTF-CWI plasmid. Data are mean ± S.E.M from a representative experiment out of 3 independent experiments performed in triplicate. (**b**) Cell surface expression of receptors, after transfection with the adjusted doses of plasmids, was determined by immunofluorescence imaging using an antibody against the N-terminal HA-tag in non-permeabilized conditions. Nuclear counterstaining with DAPI. Representative images from 3 independent experiments are shown (scale bars: 20 µm). (**c**) Cells were transfected with adjusted doses of either ∆NTF-CWI or mutant plasmids and pcDNA3.1 as control. Basal cAMP production was measured after overnight incubation with 0.5 mM IBMX in starvation media in an HTRF assay. cAMP production in nM is presented as mean ± S.E.M from a representative experiment out of 3 independent experiments performed in duplicate. Data were compared with ∆NTF-CWI with one-Way ANOVA with Dunnett’s test. ****P < 0.0001. (**d**) Cells were transfected with adjusted doses of either ∆NTF-CWI or mutant plasmids along with CRE-Luc plasmid. After an overnight of serum starvation, cells were activated with either vehicle (DMSO) or 100 µM of P-15 for 5 h. Luciferase induction was measured in a luminescence-based assay. Relative light units (RLU) recorded in a luminometer are mean ± S.E.M from a representative experiment from 3 independent experiments performed in triplicate. Data were compared between vehicle and P-15 for each plasmid with two-Way ANOVA with Sidak’s multiple comparison test. ****P < 0.0001; ns: not significant.

We also used adjusted doses of ∆NTF-CWA, ∆NTF-CAI, ∆NTF-CAA plasmids, which resulted in similar surface expression compared with ∆NTF-CWI (Fig. 6a,b). Using a homogenous time-resolved FRET (HTRF) assay in the presence of IBMX, an inhibitor of phosphodiesterases, we measured the basal cAMP production and found that while ∆NTF-CWI-expressing cells show high cAMP levels, this basal activity is absent in ECL2-mutated ∆NTF receptors (Fig. 6c). Also, stimulation with synthetic P-15 increased the CRE luciferase activity only in the ∆NTF-CWI expressing cells (Fig. 6d).

These data suggest that an intact CWI motif is essential for activation of ADGRG2 via either the self-activating *Stachel* (in ∆NTF) or the synthetic peptide. Also, residues other than CWI in the ECL2 may play a role in the activation of ADGRG2 by P-15.

### Gαs pathway activation by the Stachel is controlled by the ECL2

**Figure 7:**
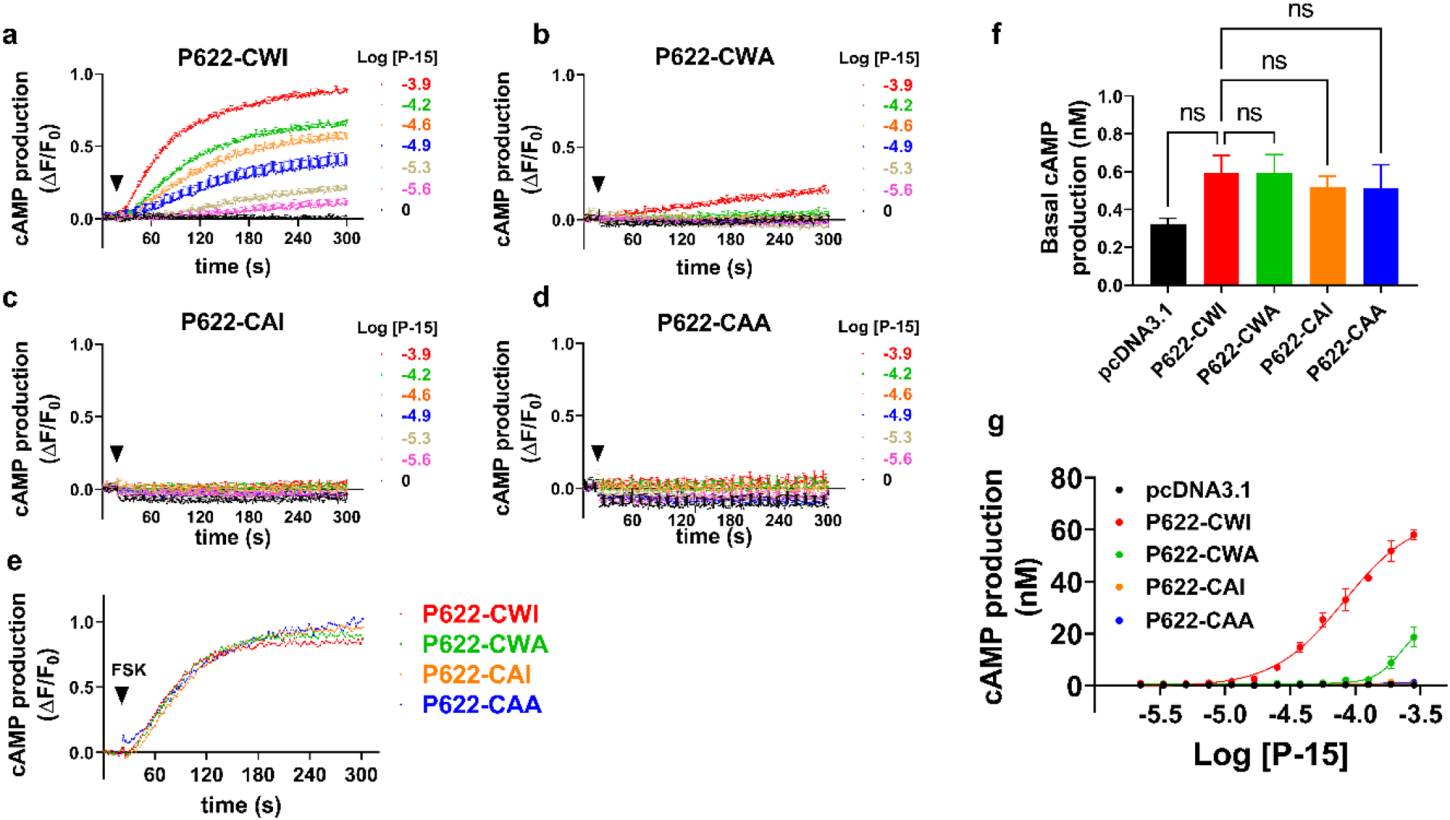
ECL2-mutated ADGRG2-P622 receptors are not activated by P-15. (**a**–**d**) Cells were transfected with adjusted doses of P622-CWI and mutant plasmids and were transduced with a cAMP biosensor overnight. After a 20-s basal recording of fluorescence (Ex: 488 nm; Em: 525 nm), cells were activated with increasing concentrations of P-15 (arrowheads), and fluorescence was recorded for another 280 s. Relative fluorescence unit (RFU) data were analyzed in GraphPad Prism and are presented as change in RFU divided by the initial RFU (ΔF/F0). (**e**) Cells transfected with adjusted doses of plasmids and biosensor were stimulated with 1 µM forskolin (FSK). Data are mean ± S.E.M and are representative of 3 independent experiments performed in triplicate. (**f**) Basal cAMP production was measured after overnight incubation with 0.5 mM IBMX in starvation media in an HTRF assay. cAMP production in nM is presented as mean ± S.E.M from a representative experiment out of 3 independent experiments performed in triplicate. Data were compared with P622-CWI with one-Way ANOVA with Dunnett’s test. ns: not significant. (**g**) Cells were stimulated with increasing concentrations of P-15 for 2 h and cAMP production in nM was measured in an HTRF assay. Data are mean ± S.E.M from a representative experiment from three independent experiments performed in duplicate.

We have previously used an endpoint HTRF assay to measure cAMP production downstream of ADGRG2-P622-Gαs pathway^11^. To monitor the production of cAMP in kinetic mode, we developed a new cAMP production assay by using a genetically-encoded biosensor^25^. We induced cAMP production by forskolin, an activator of adenylyl cyclase, and optimized the amount of sensor needed to reach a robust adenylyl cyclase response (Supplementary Fig. 4a,b). A 20-s baseline measurement was followed by stimulation with increasing concentrations of P-15 in cells that were transfected with adjusted doses of P622-CWI and ECL2-mutated receptors (Fig. 7a–d). While the P622-CWI receptor responded to P-15 in a concentration-dependent manner, the mutants showed no response, except for the P622-CWA mutant that responded marginally to the highest P-15 concentration. To ensure that increased doses of transfected plasmids did not alter the responsiveness of the adenylyl cyclase-cAMP axis, we compared the forskolin-induced cAMP production. Our data show that despite increased doses of transfected mutant plasmids, cells respond similarly to the forskolin (Fig. 7e).

We accumulated cAMP production overnight in the presence of IBMX and using an HTRF assay we found that the basal levels of cAMP are comparable among receptors (Fig. 7f). A 2-h stimulation with P-15 resulted in a concentration-dependent production of cAMP by the P622-CWI receptor (Fig. 7g) and a modest response by P622-CWA mutant.

Together, these data show that induction of the Gαs-adenylyl cyclase-cAMP pathway by *Stachel* is dependent on the presence of an intact ECL2.

### ECL2 mutations derail the whole-cell response to P-15

**Figure 8:**
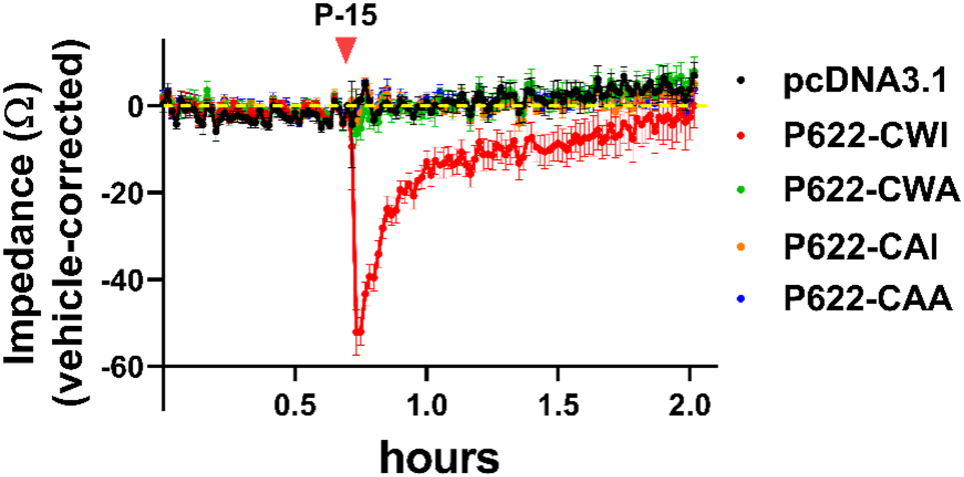
Whole-cell impedance assay shows a lack of activation of the ECL2-mutated ADGRG2 receptor by the *Stachel* peptide. Cells were transfected with adjusted doses of WT and mutant plasmids and were then seeded in plates that have electrodes at the bottom of each well to apply various frequencies of electric voltages. Serum-starved cells, at monolayer confluency, were kept in Maestro Z device until the recorded impedance (Ω) reached a steady state. Cells were stimulated with either vehicle (DMSO) or 100 µM P-15 and impedance was recorded for up to 2 h. Data are corrected to that of the vehicle for each plasmid and are mean ± S.E.M from a representative experiment out of 3 independent experiments conducted in quadruplicate.

We and others have shown that ADGRG2 couples to multiple signaling pathways including Gαq, Gαs, and Gα13^7,11,26,27^. Considering that measuring the production of either a single second messenger (e.g. cAMP) or induction of a couple of transcription factors (e.g. CRE and SRE) cannot detect the ‘overall’ response of cells to GPCR ligands, whole-cell label-free assays have been developed^28–31^. To investigate if the overall response of cells to synthetic P-15 is regulated by the ECL2 conserved residues, we developed a new non-invasive whole-cell label-free impedance assay. Activation of P622-CWI-transfected cells with P-15 resulted in a rapid decrease in cell monolayer impedance to 1 kHZ frequency of electrical field voltage (Fig. 8). However, neither empty plasmid nor ECL2-mutated receptors responded to the P-15 stimulation.

This data confirms the lack of receptor activation by *Stachel* peptide in the absence of ECL2 conserved tryptophan and isoleucine residues.

## Discussion

Our understanding of the mechanisms of activation of aGPCRs has substantially increased over the last decade. This progress was made possible due to basic pharmacology and cell biology studies in various cellular backgrounds and model organisms^8,9,32–34^. Now we know that binding of an extracellular molecular partner to the NTF of ADGRG1 dissociates it from the CTF and unmasks a *Stachel* sequence, which subsequently activates ADGRG1^14,19,35^. Similar mechanisms of activation exist for a few other aGPCRs^7,17^. A comprehensive mutational analysis of ADGRL1 revealed that several residues in the transmembrane domains regulate the constitutive activity of this aGPCR^36^. The same study also predicted a non-conserved tyrosine at the end of ECL2, which regulates *Stachel*-mediated signaling of ADGRL1. However, the binding site of the *Stachel* on a majority of aGPCRs remains largely unknown. Structural similarities of class B1 GPCRs to aGPCRs (formerly class B2) and the fact that class B1 receptors bind to peptide hormones provides hints on the possible binding site of the *Stachel*. Specifically, the ECL2 forms an orthosteric binding pocket for peptide hormones in PTH 1 receptor (PTH1R)^37^ and corticotropin-releasing factor 1 receptor (CRFR1)^38^. In the current study, we tested the role of conserved residues in and around the CW motif of the ECL2 in the activation of an NTF/*Stachel-* or NTF-truncated ADGRG2 (P622 and ∆NTF) by its *Stachel*, P-15. Our results provide evidence that the lack of isoleucine and/or tryptophan in the ECL2 not only completely abolishes the signaling pathways of ADGRG2 but also dysregulates receptor stability in HEK293 cells.

We focused on the ADGRG2-P622 receptor in this study because we had previously shown that ADGRG2-P622 responds significantly more to the synthetic P-15 in comparison to the full-length receptor (ADGRG2-FL) in a battery of assays^11^. This maximal response by ADGRG2-P622 is presumably due to the availability of the binding site for P-15, which might be masked by the NTF in ADGRG2-FL. The lack of constitutive activity in cells that express ADGRG2-P622 in the current study confirms the role of endogenous *Stachel* in the activation of ADGRG2.

Here, we used several readouts to assess receptor response to the synthetic peptide. The use of kinetic and endpoint cAMP production assays allowed us to measure the Gαs signaling of ADGRG2. Genetically encoded biosensor and HTRF assays showed a lack of activation of ECL2 mutants either in the short-term (minutes) or long-term (2 h). We supplemented these assays with CRE and SRE reporter assays, in which cells are activated for 5 h. Both assays showed that ECL2 mutants are not activated by P-15, suggesting that the coupling of both Gαs and Gα13 proteins to ADGRG2 is ablated in the absence of either tryptophan or isoleucine or both. To assess other possible but unknown signaling pathways that may originate from ADGRG2, we developed a whole-cell label-free impedance assay. We have previously used similar label-free assays to study GPR55 and cannabinoid 2 receptor^39,40^. This method allowed us to measure the overall response, rather than a singular signaling pathway, from a monolayer of cells upon activation with P-15 in kinetic mode. Using this readout, we observed a strong fast *Stachel*-specific reduction of impedance from WT receptor-expressing cells, indicating possible movement of cells and consequent disruption of cell–cell contact in the monolayer. This response was transient, and impedance was mostly restored in 15 min. Nevertheless, we did not detect a response from cells expressing any of the ECL2 mutated receptors, further confirming the lack of activation by P-15.

It is noteworthy that mutant receptors were degraded in the absence of either endogenous or synthetic *Stachel* peptide. Do the ECL2 mutations induce constitutive activity followed by sustained internalization and degradation? This does not seem to be plausible because we did not detect any altered basal activity of P622, either in CRE reporter assay or cAMP production assays. We observed a similar pattern of degradation in ECL2-mutated ∆NTF receptors; however, those mutants lacked the constitutive activity of ∆NTF, further suggesting that (1) ECL2 plays a role in the activation of ADGRG2 and (2) degradation of ECL2 mutants is not due to constitutive activity or presence of the *Stachel*. The P622-^G772A/S773A^ mutant, in which the CWI motif is intact but glycine^772^ and serine^773^ of the ECL2 are mutated, however, was not degraded. This mutant showed only 30–40% reduction in signaling to CRE and SRE, which is a much smaller effect than the mutations in the CWI motif (~ 100% reduction). This suggests that while residues of the CWI motif regulate both receptor stability and signaling, other non-conserved residues of the ECL2 contribute to ADGRG2 response to synthetic P-15 and this effect is independent of receptor stability.

Do the ECL2 mutations hinder receptor transport and incorporation into the plasma membrane? Our western blotting, ELISA, and immunofluorescence imaging data suggest that the diminished surface expression of mutants is likely to be due to the fast degradation of receptors upon translation. We have previously shown that ADGRG2-∆NTF gets ubiquitinated but is expressed on the plasma membrane at levels comparable to that of the full-length ADGRG2^11,12^. Our current data show that the degradation of P622 mutants is mediated in part by proteasomal pathways but not via endosomal or lysosomal pathways. Further detailed studies are needed to unravel how the isoleucine and tryptophan of ECL2 impact these degradation mechanisms and whether the transport of ECL2 mutants via Golgi apparatus is impaired. Interestingly, a previous study showed that many of the mutants of ADGRL1 with blunted activity have significantly reduced surface expression^36^, although the underlying cause(s) for such reduction was not explored.

Irrespective of the underlying reason for diminished receptor levels and degradation, we adjusted the surface expression levels by increasing the dose of the transfected plasmids and conducted our signaling assays in this condition. Nevertheless, comparable surface expression of ECL2-mutated and P622 or ∆NTF receptors did not rescue their response to P-15, supporting the role of ECL2 in *Stachel*-mediated activation of ADGRG2. Whether the ECL2 of other aGPCRs play similar roles in receptor stability and activation by *Stachel* peptides warrants further studies.

While finalizing this manuscript, we noticed that a separate group reported that several residues, including the tryptophan and isoleucine of the ECL2, participate in the binding of *Stachel* peptide to ADGRG2^41^. Although this study corroborates some of our findings, differences exist between the two studies. We used ‘human’ ADGRG2-P622 that lacks the NTF and the *Stachel* sequence to prevent interaction of the endogenous P-15 on the N-terminus of the receptor with the receptor in addition to ADGRG2-∆NTF. Sun et al., however, determined the binding of a ‘mouse’ P-15 that was modified at several residues to the ‘mouse’ ADGRG2 ortholog. This modified P-15 showed higher potency compared to natural P-15 and is potentially a new pharmacological tool for future ADGRG2 studies. However, its application remains to be validated by testing whether it shows affinity or activity towards other aGPCRs that share similar *Stachel* sequence and ECL2 motifs as ADGRG2. Another major difference between these two studies is that while we observed a significant alteration of ECL2-mutated receptor stability and cell surface expression, Sun et al. did not report such changes.

Lack of activation of P622 or ∆NTF by the *Stachel* could be due to either impaired binding of the peptide to the ECL2 or impaired signaling of receptor irrespective of peptide binding. Although we did not directly examine the binding affinity of the P-15 to the ECL2 mutants, authors of the recent paper^41^ showed that mutations in TM6 and ECL2 abolish the binding of a modified *Stachel* to the ADGRG2.

ADGRG2 plays various physiological and pathological roles. For instance, mutations or truncations in ADGRG2 are associated with different forms of male infertility^42,43^ and male *Adgrg2*-null mice are infertile^44^. ADGRG2 also promotes proliferation and metastasis of Ewing sarcoma cells both in vitro and in vivo^45^. We have previously shown that ADGRG2 is enriched in parathyroid glands, is upregulated in parathyroid adenomas from patients with primary hyperparathyroidism, and its activation by P-15 elevates PTH secretion^12^. Although ADGRG2 is still an orphan aGPCR, our findings on the role of ECL2 in receptor stability and *Stachel*-mediated activation provide a foundation for future targeting of this receptor via small molecules or biologics.

## Methods

### Cell culture and transfection

AD-293 (HEK) cells were purchased from Agilent Technologies (Santa Clara, CA, USA #240085) and cultured in DMEM media (Sigma, St. Louis, MO, USA #D6429) supplemented with 10% FBS, 100 U/mL penicillin, and 100 μg/mL streptomycin (Thermo Fisher Scientific, Waltham, MA, USA #15140-122). Cells were transfected with plasmids using lipofectamine 2000 reagent (Thermo Fisher Scientific #11668019) following the manufacturer’s instructions.

### Antibodies

Antibodies were purchased from the following sources: Cell Signaling Technologies (Beverly, MA, USA): rabbit anti-HA (#3724); Thermo Fisher Scientific: mouse anti-V5 (#R960-25); Sigma: rabbit anti-β-actin (#A2066); Biolegend (San Diego, CA, USA): mouse anti-HA (#901513).

### Receptor mutagenesis

We used the Q5 site-directed mutagenesis kit (NEB, Ipswich, MA #E0552S) to generate mutations in the ECL2 of human ADGRG2. Our template for mutations was pcDNA3.1-3xHA-P622-V5 (P622-CWI)^11^, a plasmid that expresses an NTF/*Stachel*-truncated ADGRG2 with an N-terminal 3xHA tag and a C-terminal V5 tag. The following primer pairs were used to construct mutants: P622-CWA^780^ (*for*: CTTCTGCTGGGCCAACAACAATGC; *rev*: TCATCCGGTGAACCATTG), P622-CA^779^I (*for*: TGACTTCTGCGCCATCAACAACAATGCAGTATTC; *rev*: TCCGGTGAACCATTGGGG), P622-CA^779^A^780^ (*for*: TGACTTCTGCGCCGCCAACAACAATGCAGTATTCTAC; *rev*: TCCGGTGAACCATTGGGG), P622-^G772A/S773A^ (mutation of G^772^ and S^773^ in the ECL2; *for*: ATTCCCCAATGCTGCTCCGGATGACTTC; *rev*: TTCCCATAGGATCCAAGC). We used the aforementioned primers and pcDNA3.1-3xHA-∆NTF-V5 (∆NTF-CWI) as the template to generate mutants of ECL2 of the NTF-truncated ADGRG2. The resulting constructs were verified by sequencing both strands.

### On-cell ELISA

Cells were seeded in 96 well plates and transfected with 50 ng of one of the following plasmids: pcDNA3.1 (empty vector), P622-CWI, P622-CWA, P622-CAI, and P622-CAA. Twenty-four hours after transfections, cells were starved using DMEM (Thermo Fisher Scientific #21068028) supplemented with glutamine and 1.25 mM Ca^2+^ overnight. Cells were then fixed with 4% paraformaldehyde for 15 min at room temperature. After several washes with TBS, cells were blocked for 30 min in TBSM (TBS + 3% milk) for surface staining or TBSM supplemented with 0.2% Triton X-100 for total staining. Cells were then incubated with either rabbit anti-HA (1:2000) or mouse anti-V5 (1:2000) antibodies in TBSB (TBS + 3% BSA) for 2 h at room temperature. After several washes, cells were incubated for 1 h with 1:2000 dilution of either horseradish peroxidase (HRP)-linked horse anti-mouse IgG (Cell Signaling Technologies #7076) or HRP-linked goat anti-rabbit IgG (Cell Signaling Technologies #7074) antibodies in TBSM. After 5 washes with TBS, cells were incubated with 3,3′,5,5′-Tetramethylbenzidine (TMB) (Sigma #t0440) for 5 min at room temperature. The reaction was stopped by an equal volume of 1 N HCl. Absorbance at 450 nm was measured using a FlexStation III plate reader. In a separate set of experiments, cells were transfected with several amounts of plasmids to determine the doses of mutant plasmids that result in similar surface expression compared to non-mutated P622-CWI or ∆NTF-CWI receptors.

### Degradation assay

Cells were transiently transfected with 1 µg of plasmids in 6-well plates and 24 h post-starvation were incubated with 100 µg/mL of cycloheximide (Sigma #4859) for indicated time points. In some experiments, transfected cells were pre-incubated with 25 µM chloroquine (Tocris, Minneapolis, MN, USA #4109), 1 µM MG132 (Cell Signaling Technologies #2194), or 10 nM bafilomycin A1 (Tocris #1334) for 16 h before the 8-h cycloheximide treatment. Cells were lysed using radioimmunoprecipitation assay (RIPA) lysis buffer (EMD Millipore, Billerica, MA, USA #20-188) supplemented with cocktails of protease (Thermo Fisher Scientific #78429) and phosphatase inhibitors (EMD Millipore #524,625). Lysates were then centrifuged at 13,000 rpm for 5 min at 4 °C and supernatants were collected.

### Western blotting

Protein lysates were boiled with reducing sample buffer (Thermo Fisher Scientific #NP0007 and #NP0009) for 10 min and loaded on 4–12% Bis–Tris gels (Thermo Fisher Scientific #NP0336BOX) then transferred to polyvinylidene fluoride (PVDF) membranes. Blocking was performed in TBSTM (TBS + 0.1% Tween-20 + 10% nonfat dry milk) followed by incubation with primary antibodies in TBSTM (1:2000 for HA and V5; 1:500 for β-actin), overnight at 4 °C. To detect primary antibodies, cells were incubated with HRP-linked horse anti-mouse IgG and HRP-linked goat anti-rabbit IgG antibodies (1:5000) in TBSTM for 2 h at room temperature. Blots were washed and then developed with ECL SuperSignal™ West Femto substrate (Thermo Fisher Scientific #34095). Blots were imaged separately via an auto-exposure option that avoids saturation of any bands and were then analyzed in iBright™ FL1500 Imaging System (Thermo Fisher Scientific).

### Immunofluorescence staining and imaging

HEK cells were transfected with plasmids (either 50 ng or adjusted doses as stated in each figure), seeded on glass coverslips, and serum-starved overnight. Cells were fixed in 4% paraformaldehyde for 10 min and were washed with PBS several times. Cells were blocked with 5% goat serum in PBS for 1 h and incubated with mouse anti-HA antibody (1:1000) in 1% BSA in PBS for 2 h at room temperature. After several washing steps with PBS, HA antibody-bound receptors were labeled with Alexa Fluor 594–conjugated goat anti-mouse antibody (1:500) in 1% BSA in PBS for 1 h. Cells were mounted in ProLong Diamond Antifade Mountant with DAPI (ThermoFisher Scientific #P36971) for nuclear counterstaining. Fluorescence microscopy was conducted by 40 × oil objective (1.4 NA) on a Nikon Ti-E microscope equipped with a 16.2 MegaPixels DS-Ri2 camera and images were analyzed with Nikon NIS-Elements Basic Research software.

### Luciferase reporter assays

Luciferase reporter assay was performed as described previously^12^, with some modifications. HEK cells were seeded in white clear-bottom 96-well plates (20,000 cells/well) and were transfected with adjusted amounts of receptor plasmids along with 100 ng of either pCRE-Luc or pSRE-Luc reporter plasmids. Cells were then stimulated with increasing concentrations of P-15 or vehicle for 5 h at 37 °C. Using Steadylite reagents (PerkinElmer, Hopkinton, MA, USA, #6066756), the luminescence was measured in a FLEXStation III device.

### cAMP production assays

Cells were resuspended in DMEM + 10% FBS and were mixed with Upward Green cADDis cAMP Assay Kit BacMam sensor (Montana Molecular, Bozeman, MT, USA #U0200G), supplemented with 2 mM sodium butyrate. Cells were then seeded at 50,000 cells/well in black, clear-bottom, 96-well plates (150 µl/well), incubated for 30 min at room temperature in the dark, and then at 37 °C for 24 h. Cells were washed with assay buffer (HBSS; Thermo Fisher Scientific #14065056 supplemented with 20 mM HEPES) and kept in dark to acclimate to room temperature for 1 h in 100 µl/well of assay buffer. Initial assay development was conducted by transducing with various amounts of the BacMam Gαs sensor followed by stimulation with 10 µM forskolin for 800 s (Supplementary Fig. 4). Considering the speed and amplitude of the signal, we chose 15 µl per well of the sensor as the appropriate volume to monitor cAMP production in HEK cells. After transfection with adjusted doses of plasmids, cells were transduced with 15 µl of the sensor (as described above) and were stimulated with increasing concentrations of P-15 the next day. cAMP production was recorded for 280 s after an initial 20 s of basal measurement of fluorescence at Excitation 488 nm and Emission 525 nm using the FLEXStation III plate reader. In a different set of experiments, a previously described homogenous time-resolved FRET (HTRF) assay^11^ was used to measure cAMP production either at basal condition (overnight incubation with 0.5 mM IBMX; Sigma #I5879) or after 2 h of activation with P-15 in 384-well plates.

### Whole-cell label-free impedance assay

Cells were transfected with adjusted amounts of plasmids and were seeded at a density of 50,000 cells per well in 100 µl of DMEM (Thermo Fisher Scientific #21068028) supplemented with glutamine and 1.25 mM Ca^2+^ in CytoView-Z 96-well plates (Axion BioSystems, Atlanta, GA, USA #Z96-IMP-96B) overnight. After 45 min of baseline recording of impedance in the Maestro Z device (Axion BioSystems) cells were activated with 100 µM P-15 or vehicle (DMSO). Impedance against 1 kHz voltage frequency was recorded for several hours at 37 °C, 5% CO_2_ in a humidified environment in the Maestro Z machine. Data normalization to vehicle and analyses were performed in AxIS Z software.

### Statistical analysis

Statistical analyses were conducted using t-test, one-Way or two-Way ANOVA with Dunnett’s or Holm-Sidak multiple comparison tests in GraphPad Prism 9.0 software; P < 0.05 was considered significant.

## Supplementary Information

Supplementary Information.
