# Supplementary material for: Conserved residues in the extracellular loop 2 regulate Stachel-mediated activation of ADGRG2
Source: Sci Rep. 2021 Jul 7;11:14060. doi: 10.1038/s41598-021-93577-y (PMC8263569; doi:10.1038/s41598-021-93577-y)

Supplementary Information

Conserved residues in the extracellular loop 2 regulate *Stachel*-mediated activation of ADGRG2

Abanoub A. Gad[1,2], Pedram Azimzadeh[1], Nariman Balenga[1,3, 4] *

From the [1]Department of Surgery, University of Maryland School of Medicine, Baltimore, MD, [2]Graduate Program in Life Sciences, University of Maryland, Baltimore, MD, [3]Department of Pharmacology, University of Maryland School of Medicine, Baltimore, MD, [4]Molecular and Structural Biology program at University of Maryland Marlene and Stewart Greenebaum NCI Comprehensive Cancer Center, Baltimore, MD

*To whom correspondence should be addressed: Nariman Balenga; Department of Surgery and Pharmacology, University of Maryland School of Medicine, 655 W. Baltimore Street, Room 10-027, Baltimore, MD 21201; Tel.:410-706-3261; Fax.:410-706-3260. E-mail: nbalenga@som.umaryland.edu

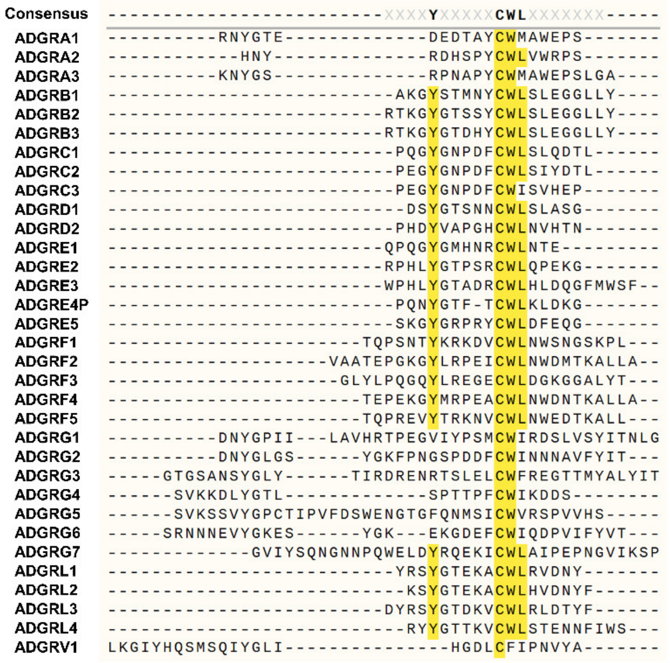

Supplementary Figure 1. Multiple alignments of the ECL2 of all 33 members of the aGPCR family. Predicted amino acid sequences of the ECL2 for each aGPCR were derived from Uniprot. The alignment was conducted in SnapGene software (from Insightful Science; available at www.snapgene.com) using the Clustal Omega algorithm.

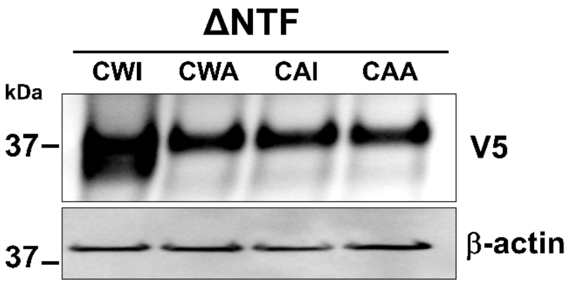

Supplementary Figure 2. HEK cells were transfected with the same dose of plasmids (1 μg) expressing ΔNTF-CWI or mutants. Total cell lysates were run on SDS-PAGE, transferred to PVDF membranes, and probed with V5 and β-actin antibodies. Representative blots (from 3 independent experiments) show differential levels of mutant receptors compared with ΔNTF-CWI. Uncropped blots are provided in Supplementary Figure 5.

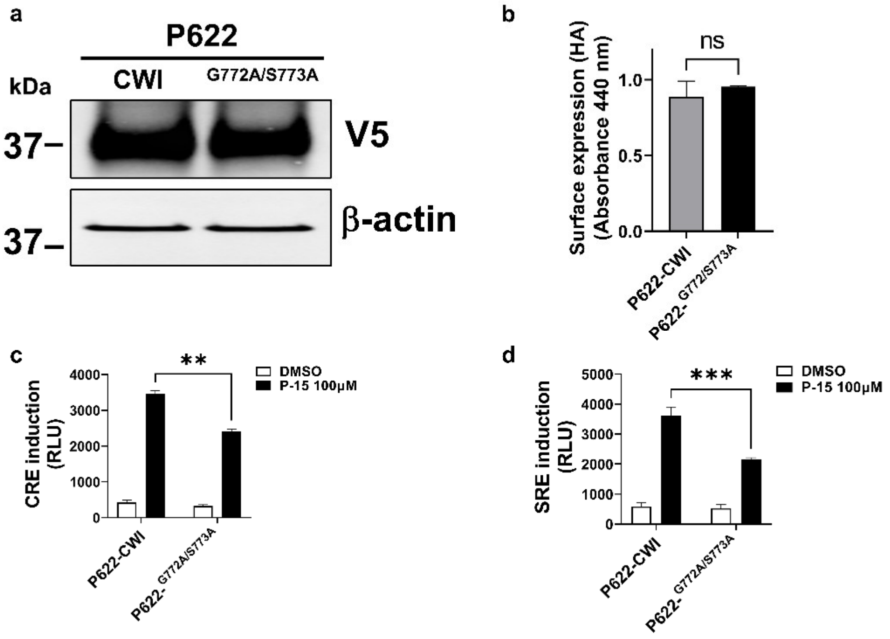

Supplementary Figure 3. (a) HEK cells were transfected with the same dose of plasmids (1 μg) expressing P622-CWI ($G^{772}S^{773}$) or mutant (P622-$^{G772A/S773A}$). Total cell lysates were run on SDS-PAGE, transferred to PVDF membranes, and probed with V5 and β-actin antibodies. Representative blots (from 3 independent experiments) show similar levels of receptors. Uncropped blots are provided in Supplementary Figure 5. (b) HEK cells were transfected with the same dose (50 ng) of plasmids expressing P622-CWI ($G^{772}S^{773}$) or mutant (P622-$^{G772A/S773A}$). Cell surface expression of receptors was determined by ELISA using an antibody against the N-terminal HA-tag in non-permeabilized conditions. Data are mean ± S.E.M from a representative experiment out of 3 independent experiments performed in triplicate. t-test was used for statistical analyses; ns: not significant. (c-d) Cells were transfected with 50 ng of either P622-CWI ($G^{772}S^{773}$) or mutant (P622-$^{G772A/S773A}$) plasmids along with either CRE-Luc or SRE-Luc plasmids. After an overnight of serum starvation, cells were activated with either DMSO or 100 μM of P-15 for 5 hours. Luciferase induction was measured in a luminescence-based assay. Relative light units

(RLU) recorded in a luminometer are mean ± S.E.M from a representative experiment from 3 independent experiments performed in duplicate. Data were compared with P622-CWI with two-Way ANOVA with Sidak's multiple comparison test. **:$P<0.01$; ***:$P<0.001$.

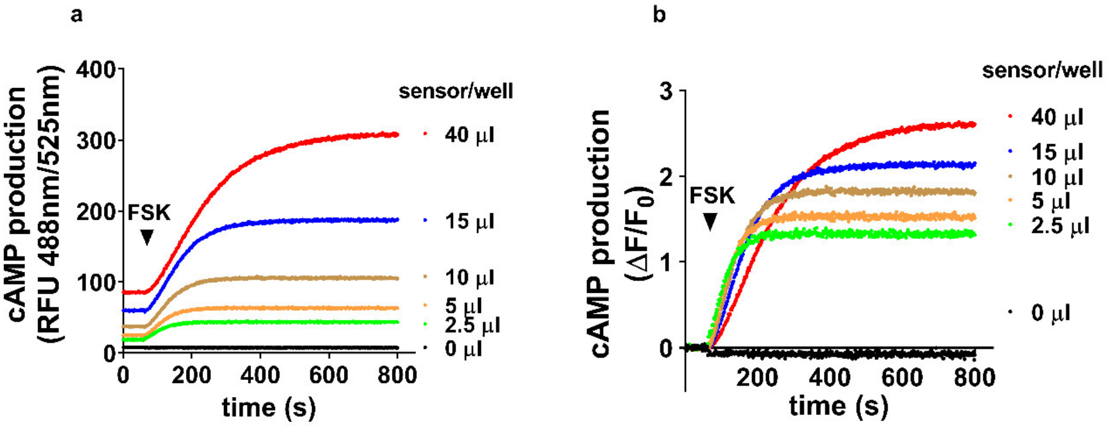

Supplementary Figure 4. Characterization of a fluorescent genetically encoded cAMP sensor. HEK cells were transduced with various amounts of Upward Green cADDis cAMP sensor in 96-well plates overnight. Cells were washed with assay buffer and then the fluorescence (Excitation at 488 nm/Emission at 525 nm) was recorded for 1 min before and 12 min after addition of 10 μM FSK. (a) Raw data showing the relative fluorescence units (RFU) for each amount of sensor. (b) Data were analyzed in GraphPad Prism and are presented as change in RFU divided by the initial RFU (ΔF/F0).

Supplementary Figure 5. Uncropped blots related to figures 2d, 3, 4, supplementary figures 2 and 3a are provided. Red boxes show the cropped images. The upper bands in the V5 blots are ubiquitinated forms of the receptors. Auto-exposure in the iBright imaging system was used for unbiased handling of western blot images.

Uncropped blots Figure 2d

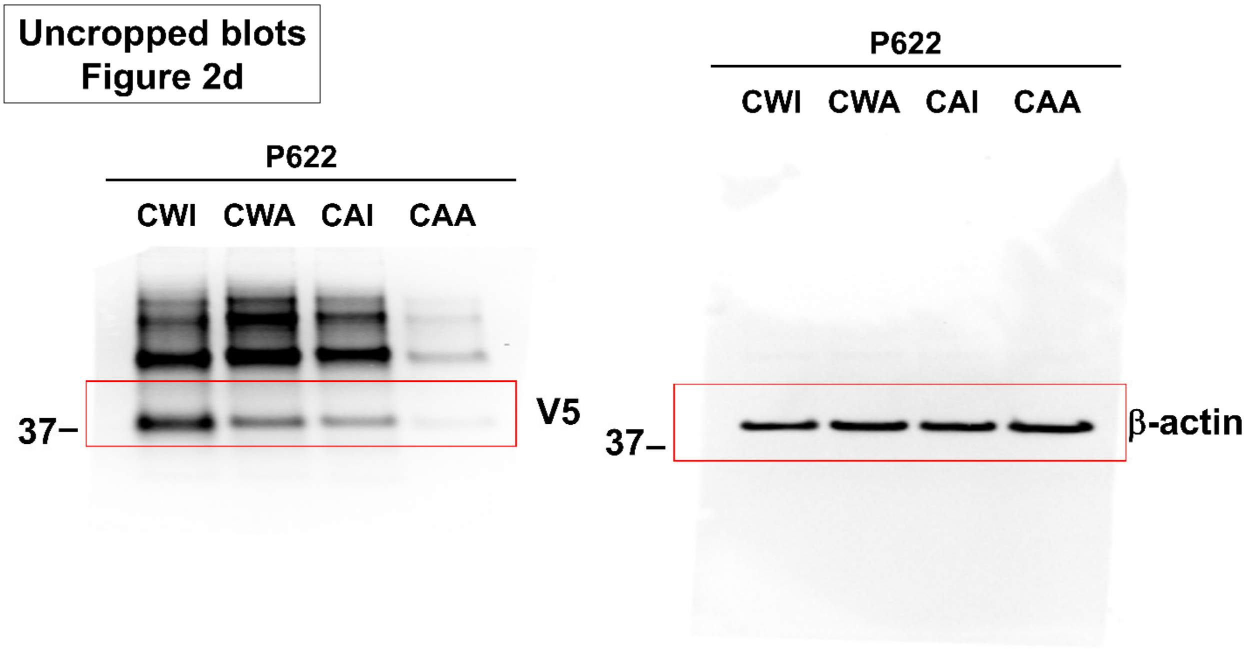

Uncropped blots Figure 3

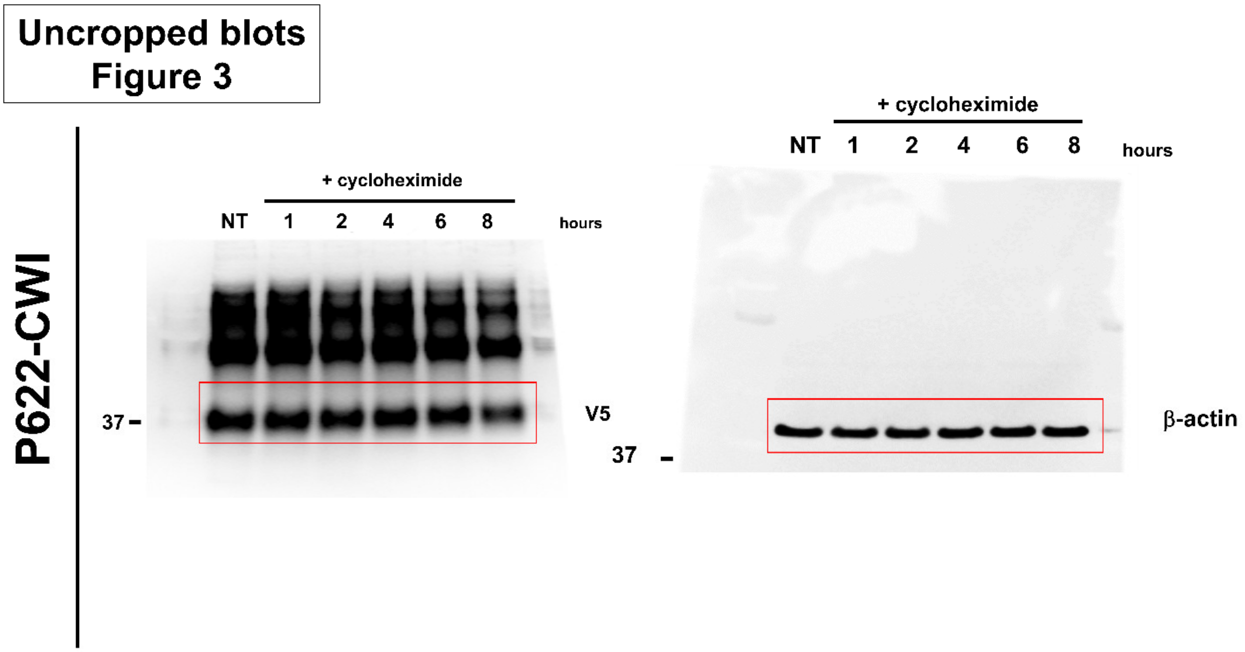

Uncropped blots
Figure 3

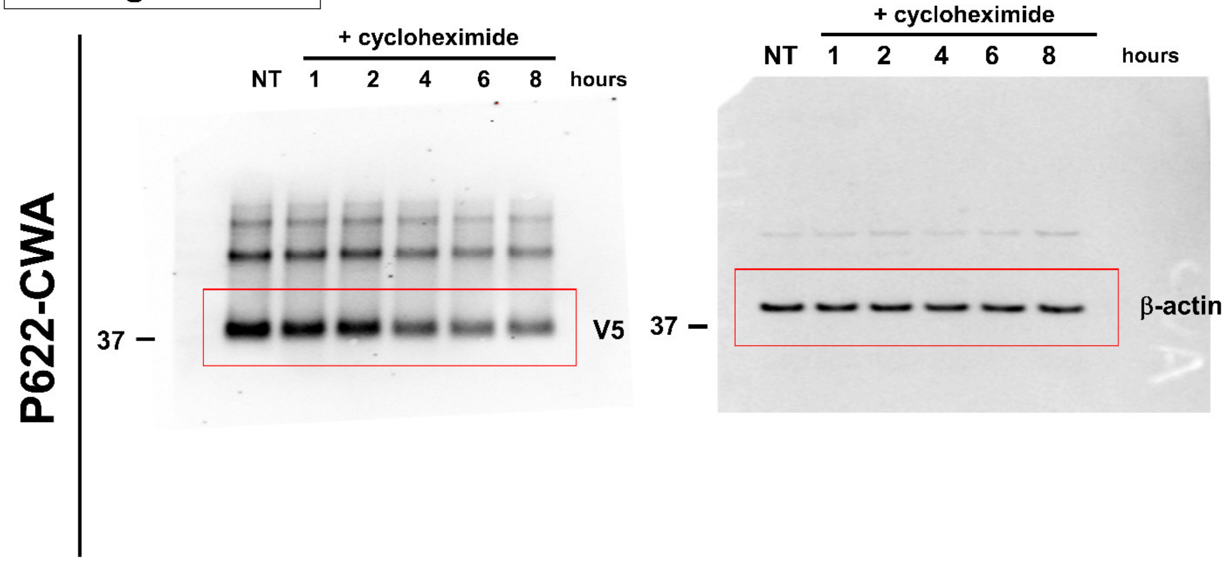

Uncropped blots
Figure 3

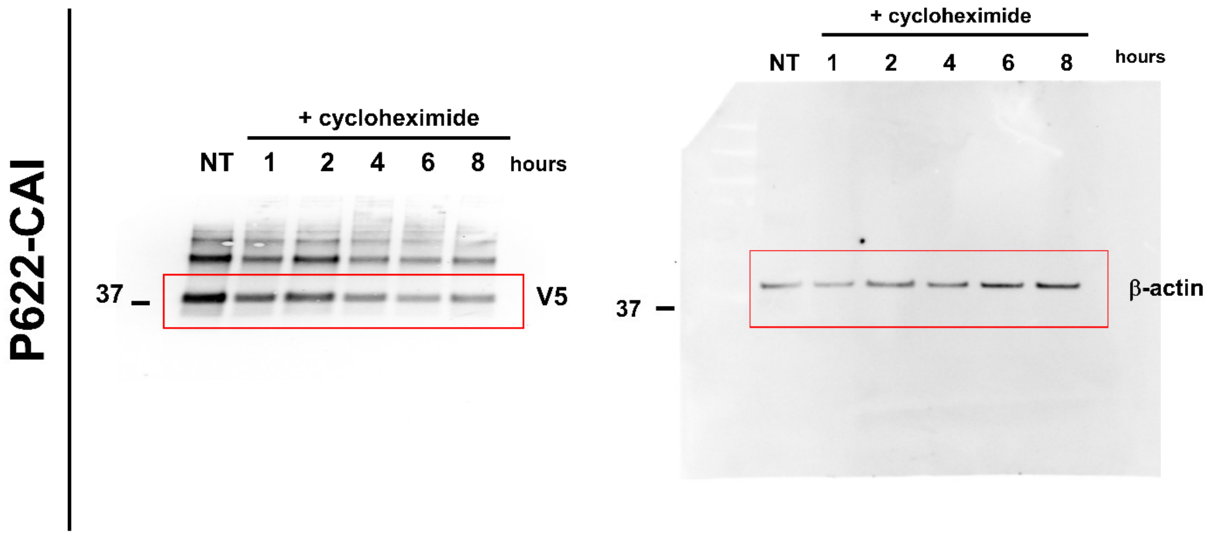

Uncropped blots
Figure 3

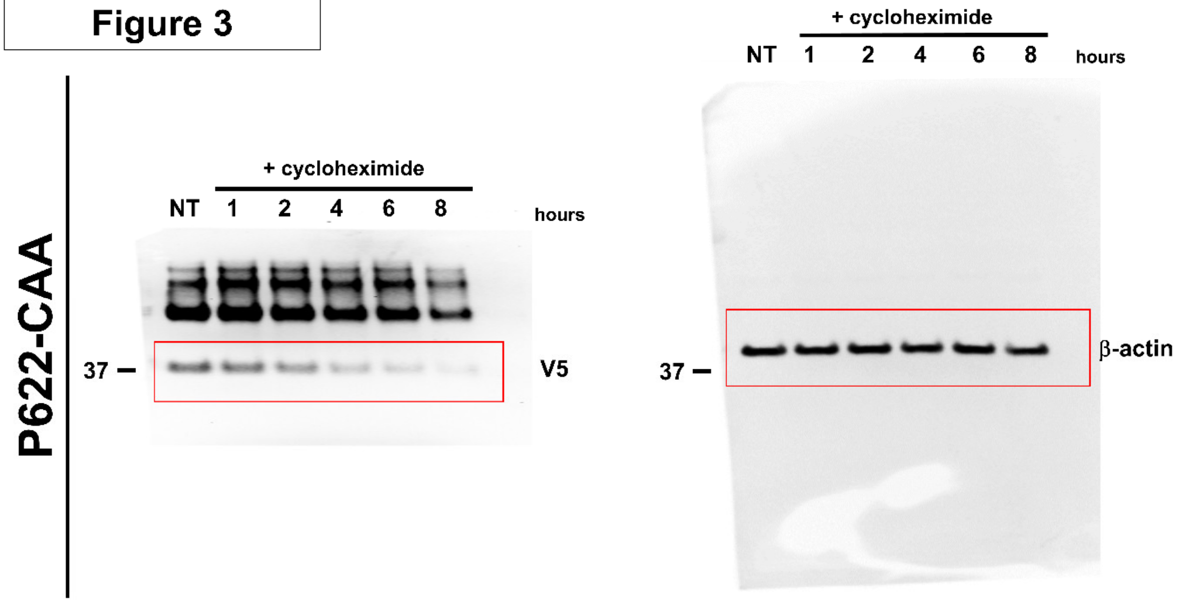

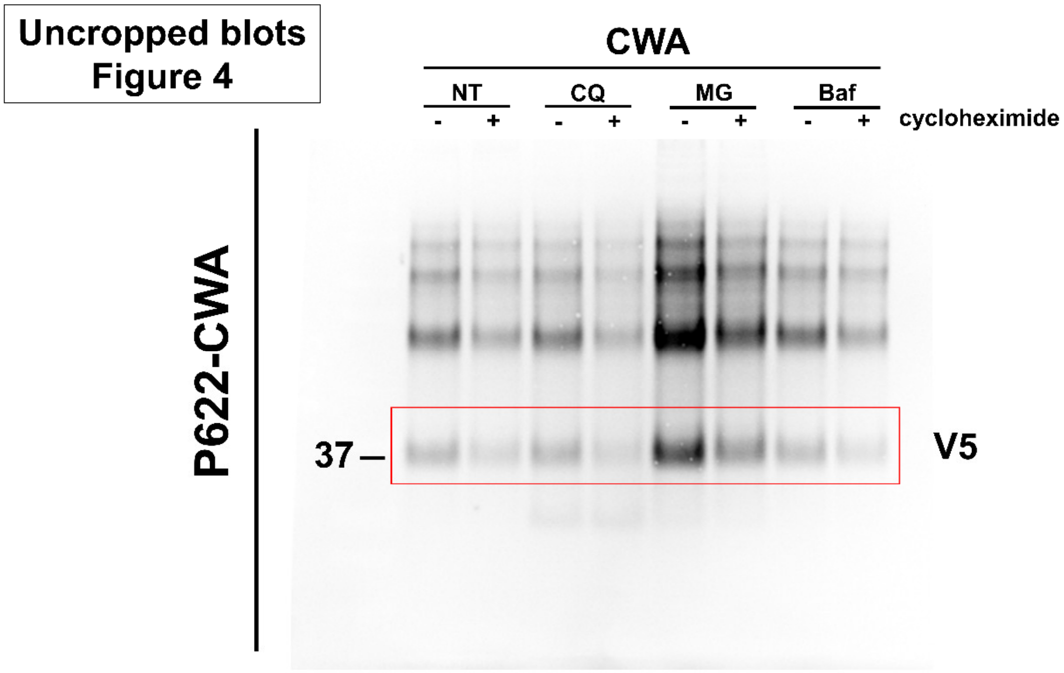
Uncropped blots
Figure 4
CWA
NT
CQ
MG
Baf
- + - + - + - +
cycloheximide
P622-CWA
37
V5

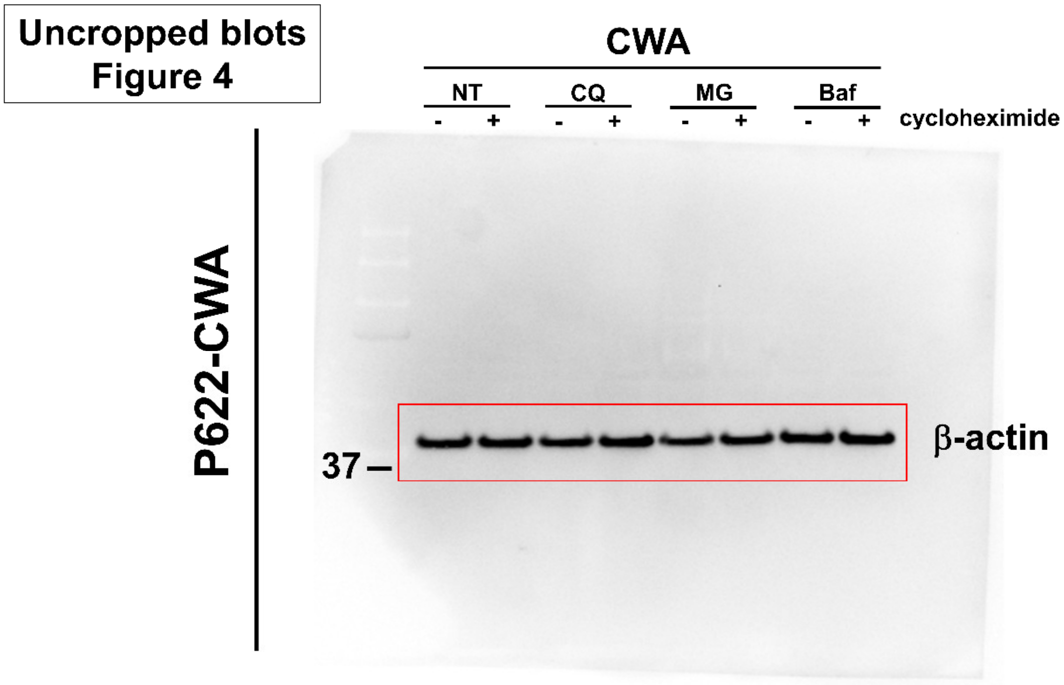
Uncropped blots
Figure 4
CWA
NT
CQ
MG
Baf
- + - + - + - +
cycloheximide
P622-CWA
37
β-actin

Uncropped blots
Figure 4

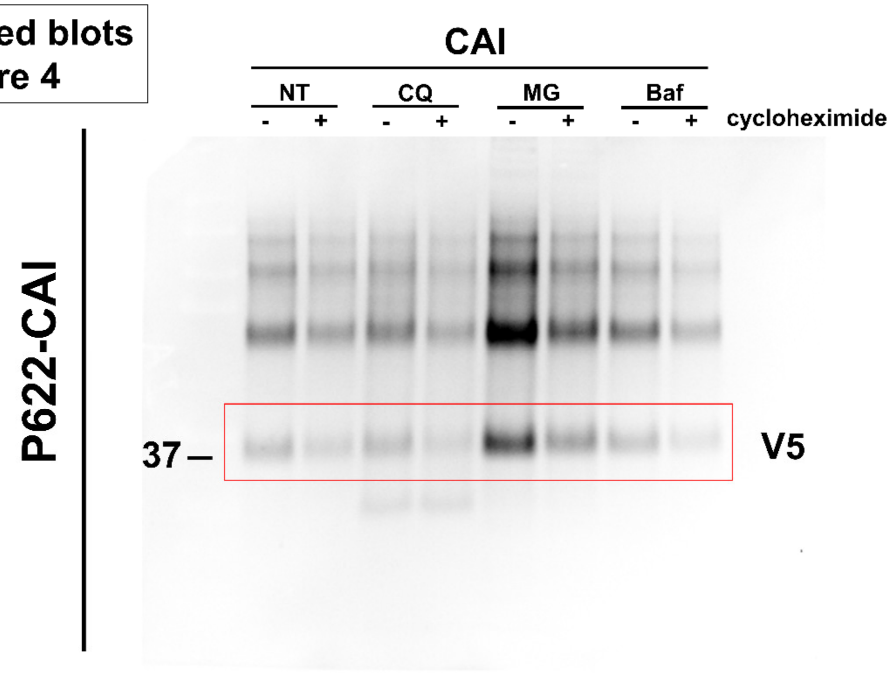

Uncropped blots
Figure 4

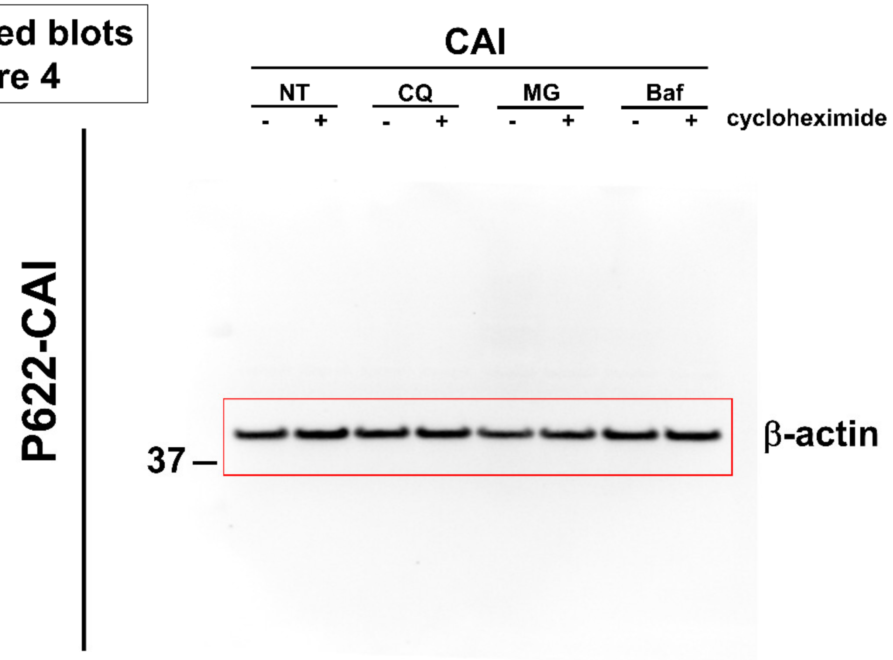

Uncropped blots
Figure 4

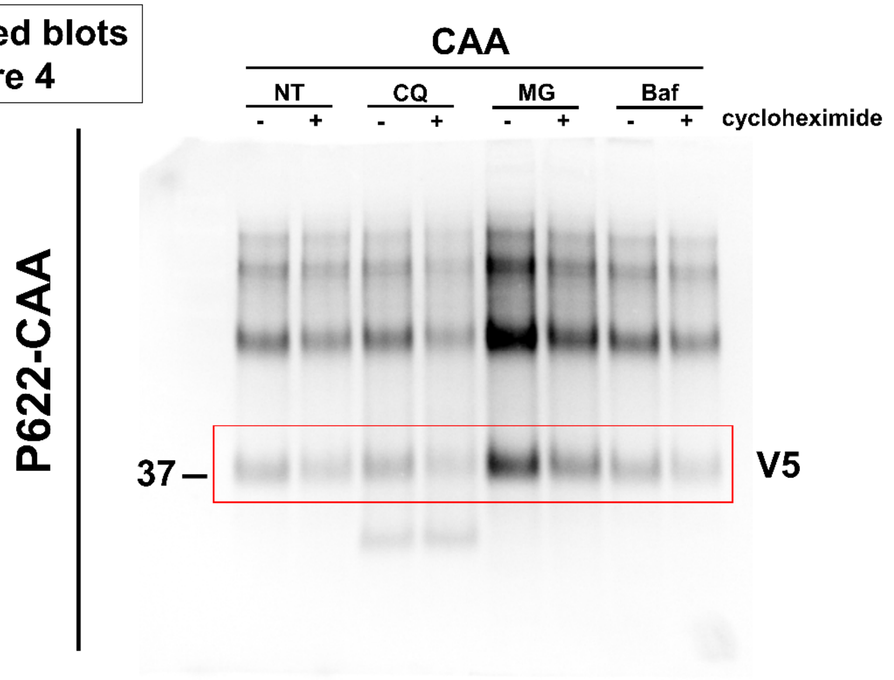

Uncropped blots
Figure 4

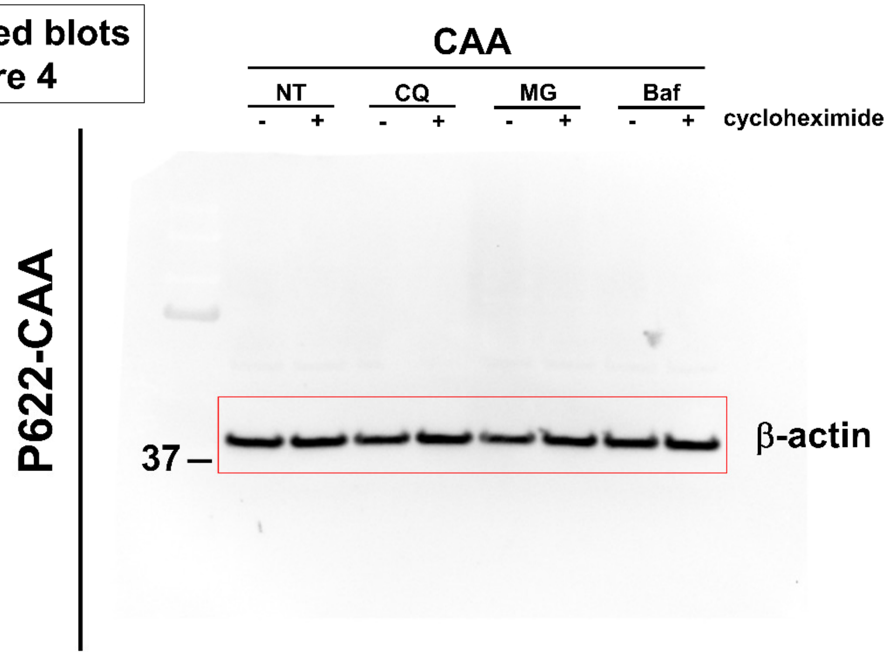

Uncropped blots
Suppl Figure 2

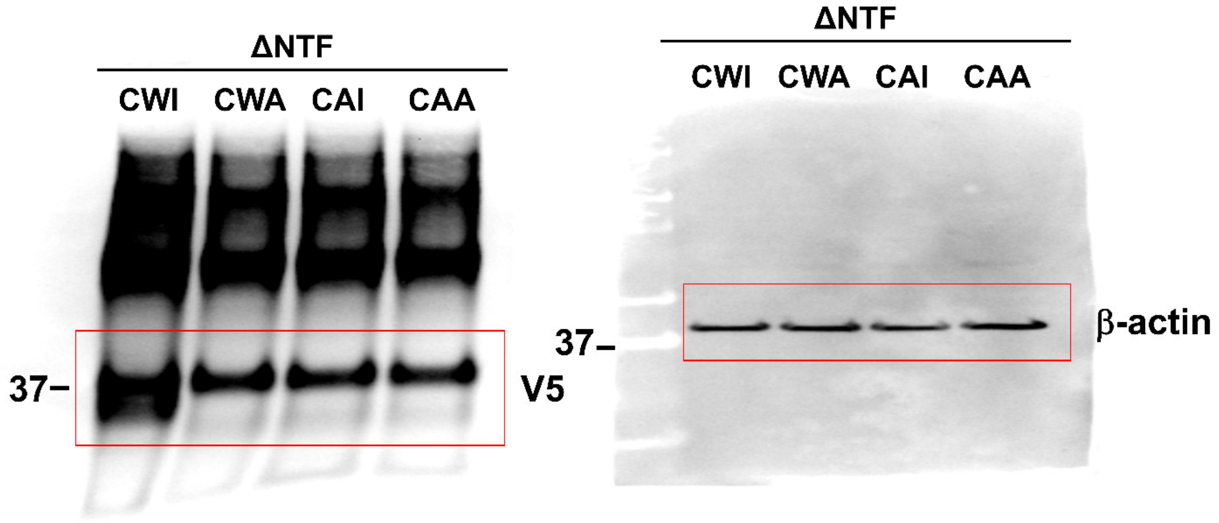

Uncropped blots
Suppl Figure 3a

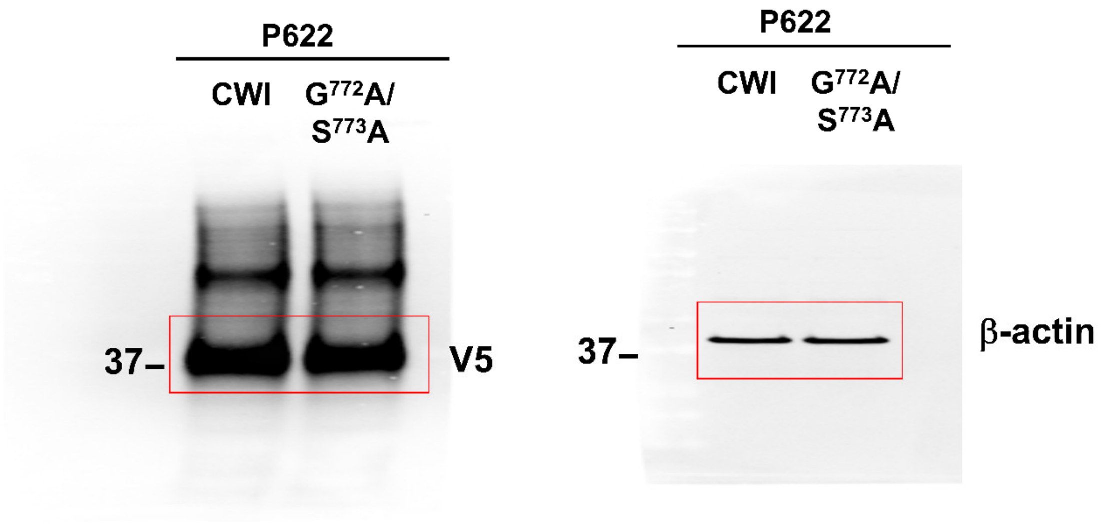

Supplement: Supplementary file 1 — Supplementary Information. [file 41598_2021_93577_MOESM1_ESM.pdf]
